# Phage-Borne Depolymerases Decrease *Klebsiella pneumoniae* Resistance to Innate Defense Mechanisms

**DOI:** 10.3389/fmicb.2018.02517

**Published:** 2018-10-23

**Authors:** Grazyna Majkowska-Skrobek, Agnieszka Latka, Rita Berisio, Flavia Squeglia, Barbara Maciejewska, Yves Briers, Zuzanna Drulis-Kawa

**Affiliations:** ^1^Department of Pathogen Biology and Immunology, Institute of Genetics and Microbiology, University of Wrocław, Wrocław, Poland; ^2^Laboratory of Applied Biotechnology, Department of Biotechnology, Ghent University, Ghent, Belgium; ^3^Institute of Biostructure and Bioimaging, Italian National Research Council, Naples, Italy

**Keywords:** bacteriophage, *Klebsiella* sp., capsule, polysaccharide depolymerase, innate immunity

## Abstract

*Klebsiella pneumoniae* produces capsular polysaccharides that are a crucial virulence factor protecting bacteria against innate response mechanisms of the infected host. Simultaneously, those capsules are targeted by specific bacteriophages equipped with virion-associated depolymerases able to recognize and degrade these polysaccharides. We show that *Klebsiella* phage KP32 produces two capsule depolymerases, KP32gp37 and KP32gp38, with a high specificity for the capsular serotypes K3 and K21, respectively. Together, they determine the host spectrum of bacteriophage KP32, which is limited to strains with serotype K3 and K21. Both depolymerases form a trimeric β-structure, display moderate thermostability and function optimally under neutral to alkaline conditions. We show that both depolymerases strongly affect the virulence of *K. pneumoniae* with the corresponding K3 and K21 capsular serotypes. Capsule degradation renders the otherwise serum-resistant cells more prone to complement-mediated killing with up to four log reduction in serum upon exposure to KP32gp37. Decapsulated strains are also sensitized for phagocytosis with a twofold increased uptake. In addition, the intracellular survival of phagocytized cells in macrophages was significantly reduced when bacteria were previously exposed to the capsule depolymerases. Finally, depolymerase application considerably increases the lifespan of *Galleria mellonella* larvae infected with *K. pneumoniae* in a time- and strain-dependent manner. In sum, capsule depolymerases are promising antivirulence compounds that act by defeating a major resistance mechanism of *K. pneumoniae* against the innate immunity.

## Introduction

With the emergence of both hypervirulent and multidrug-resistant strains, *Klebsiella pneumoniae* infections have now become a serious global health concern, especially threatening the health of hospitalized and immune-compromised patients (Podschun and Ullmann, 1998; Paczosa and Mecsas, 2016; Martin and Bachman, 2018). Most *K. pneumoniae* strains are enveloped by a thick capsular polysaccharide (CPS), which plays an important role in processes critical for the pathogenicity of these bacteria. Based on CPS diversity 78 serologically distinct capsule variants (K-types) of *K. pneumoniae* have been described (Pan et al., 2013). However, this number is certainly an underestimation of this diversity as large genomics studies have described 134 capsular K-types, which are easily transferable by homologous recombination between *K. pneumoniae* strains (Wyres et al., 2016). The serotype-associated differences in the capsule thickness and its glycan composition affect *Klebsiella* virulence (Cryz et al., 1986), e.g., higher amounts of produced CPS relate to a higher virulence (Nassif and Sansonetti, 1986). Moreover, *Klebsiella* capsule structure may lack some glyco-epitopes that could be recognized by components of the host’s innate defense, including the mannose-receptor (MR) of macrophages, mannose-binding lectin (MBL) or lung surfactants proteins (Sahly et al., 2008). In addition, capsule loss or alteration of both the composition and amount of produced CPS, either by knocking out any gene necessary for its production or by treatment with capsule-degrading agents, can lead to attenuation of virulence (Yoshida et al., 2000; Cortés et al., 2002; Lawlor et al., 2005).

Due to its external position, the CPS modulates the pathogen-host interface, both in the context of the interactions between the bacterium and the human immune system, and between the phage and the bacterium as a host. By masking underlying cell surface structures, CPS allows the bacterial cell to hide from the host defense system and evade its destructive power, which is crucial in the establishment of infection. Indeed, poorly capsulated *K. pneumoniae* strains or capsule-deficient mutants are more efficiently phagocytized by macrophages and neutrophils than heavily capsulated and wild-type strains, respectively (Álvarez et al., 2000; Cortés et al., 2002; Lawlor et al., 2005; Regueiro et al., 2006; March et al., 2013). Furthermore, CPS covering the *O*-antigen of lipopolysaccharide (LPS) or the outer membrane proteins (OMPs) as well as the other antibody epitopes on the bacterial cell surface, can also suppress the complement cascade activation, thereby preventing C3b deposition and membrane attack complex (MAC) formation (Merino et al., 1992; Albertí et al., 1996).

Phages are commonly found in the biosphere and as natural bacterial predators control its abundance in all kind of environments (marine, freshwater, soil, microbiome of multicellular organisms). On the other hand, bacteria as natural prey of phages have developed several strategies to prevent phage infection or to diminish its consequences (Olszak et al., 2017).

One of phage-resistance mechanism is to inhibit virion adsorption to bacterial surface by the production of polysaccharide thick layer such as capsule, slime or biofilm matrix. Thus besides evasion of phagocytic- and complement-mediated killing, CPS serve bacteria for the protection against viral infection. Nevertheless, some phages developed a specific strategy to overcome this barrier. To gain access to the membrane and to trigger DNA ejection, *Klebsiella* phages first recognize, bind to and enzymatically degrade CPS of bacterial cell, using virion-associated proteins with depolymerization activity. These structural enzymes, called capsule depolymerases, are mainly found in tail fibers or tail spikes, structures that also determine the phage host range (Latka et al., 2017). Phages have evolved various capsule depolymerases, providing them with diverse bacteria specificities. For instance, nine functional capsule depolymerase-encoding genes were identified in the genome of *Klebsiella* giant phage K64-1 with each depolymerase able to degrade a different CPS type (K1, K11, K21, K25, K30/K69, K35, K64, KN4, and KN5), (Pan et al., 2015, 2017). In turn, the closely related podoviruses K5-2 and K5-4 encode tail fiber proteins with K30/K69 and K5, or K8 and K5 depolymerase activities, respectively (Hsieh et al., 2017). In another study, two genes encoding putative CPS depolymerases were identified in podovirus KpV41 (Solovieva et al., 2018).

With the exhausting number of bacterial targets that could be the basis for novel antibiotic drug development, the interest in antivirulent agents including phage-encoded enzymes, has emerged as a promising strategy for infection control (Majkowska-Skrobek et al., 2016; Olszak et al., 2017; Maciejewska et al., 2018). Unlike conventional antibiotics that kill the bacterial cells or inhibit their growth, the capsule depolymerases are aimed at disarming the pathogen. Modification of the bacterial phenotype makes the cells less virulent and more susceptible to antimicrobials or innate host defense (Bansal et al., 2014; Lin et al., 2014; Pan et al., 2015). In this paper, we identified two proteins encoded by *Klebsiella* phage KP32 (*Podoviridae*, KP32 virus), the tail spike and a previously hypothetical protein that are able to degrade two different capsular serotypes. We analyzed their effect on *K. pneumoniae* virulence including the capsule-mediated resistance to complement action and phagocytosis by human macrophages as well as the susceptibility to the insect innate immune response in *Galleria mellonella* infection model.

## Materials and Methods

### Phage, Bacterial Strains, and Growth Conditions

Phage vB_KpnP_KP32 (KP32; PCM F/00068) was originally isolated from environmental water in Poland and characterized in our previous paper (Kȩsik-Szeloch et al., 2013). The *Klebsiella pneumoniae* strains used in this study (Supplementary Table S1) are provided by the Institute of Genetics and Microbiology Bacterial Collection (Wroclaw, Poland). *Klebsiella* strains were routinely cultured in Trypticase Soft Broth or Agar (TSB or TSA, BioMérieux, Marcy-l’Etoile, France) at 37°C. Unless indicated otherwise, the overnight cultures were diluted 1/100 in fresh TSB and incubated for 2 h at 37°C with shaking to log-phase. Then the cultures were harvested (5,000 × *g*, 20 min, 4°C) and resuspended to an OD_600_ of 1.0 in PBS (∼10^9^ CFU/ml). Capsular serotype was determined by sequencing of *wzi* or *wzc* genes as described elsewhere (Brisse et al., 2013; Pan et al., 2013). *Escherichia coli* TOP10 F’ and BL21(DE3) (Invitrogen, Carlsbad, CA, United States) were used for plasmid propagation and recombinant protein expression, respectively. *E. coli* strains were cultivated in Lysogeny Broth (LB, BioCorp, Lublin, Poland) at 37°C supplemented with 100 μg/ml ampicillin. All bacteria are listed in Supplementary Table S1.

### Recombinant Protein Preparation and Characterization

The open reading frames of phage KP32 DNA encoding *gp37* (YP_003347555), and *gp38* (YP_003347556) were amplified by PCR using Pfu DNA polymerase (Thermo Fisher Scientific, Lithuania) and specific primer pairs (Supplementary Table S1), and then cloned into the pEXP5-NT/TOPO^®^ and pEXP5-CT/TOPO^®^ expression vectors (Invitrogen, Carlsbad, CA, United States), respectively. Following verification by DNA sequencing (Genomed, Warsaw, Poland), *E. coli* BL21(DE3) was transformed with the correct plasmids, and overproduction of the recombinant proteins in LB supplemented with 100 μg/ml ampicillin was induced by the addition of isopropyl-β-D-thiogalactopyranoside (IPTG) to a final concentration of 0.1 mM for 18 h at 20°C. The cells were harvested (5,000 × *g*, 20 min, 4°C), resuspended in lysis buffer [300 mM NaCl, 20 mM Tris-HCl, 10 mM imidazole, 5% (v/v) glycerol, pH 7.8] containing complete protease inhibitor cocktail (Roche Diagnostics, Mannheim, Germany), and were lysed by freezing/thawing and sonication. Recombinant His-tagged proteins were then purified from the soluble fractions of the whole-cell lysates by affinity chromatography using Bio-Scale Mini Profinity IMAC cartridges (Bio-Rad, Hercules, CA, United States) in combination with a FPLC-system (Bio-Rad, Hercules, CA, United States) as previously described (Majkowska-Skrobek et al., 2016). Proteins were dialyzed against phosphate-buffered saline (PBS) buffer (137 mM NaCl, 2.7 mM KCl, 10 mM Na_2_HPO_4_, 1.8 mM KH_2_PO_4_, pH 7.4) using Float-A-Lyzer (Serva, Heidelberg, Germany), and analyzed by 12% SDS-PAGE according to the method of Laemmli (1970) and size-exclusion chromatography (SEC) coupled to a miniDAWN TREOS multi-angle static light scattering (MALS) detector (Wyatt Instrument Technology Corp., Santa Barbara, CA, United States) and an Optilab^TM^ rEX (Wyatt Instrument Technology Corp.). Circular dichroism studies were carried out as described previously (Majkowska-Skrobek et al., 2016). Protein concentrations were measured fluorometrically (Qubit 2.0, Invitrogen, Carlsbad, CA, United States) or spectrophotometrically (NanoDrop, 2000 Spectrophotometer, Thermo Fisher Scientific) using a molar extinction coefficient of 107,730 cm^−1^⋅M^−1^ (for KP32gp37) and 63,620 cm^−1^⋅M^−1^ (for KP32gp38) estimated by ProtParam^1^. Visualization of phage KP32 and recombinant depolymerases specificities on bacterial lawns was performed using a standard spot assay (Majkowska-Skrobek et al., 2016).

### Depolymerase Stability Measurement

To estimate the pH-dependent stability, depolymerase was suspended at the desired pH, using 50 mM CH_3_COONa-HCl buffer (pH 3.0–5.0), 50 mM NaH_2_PO_4_-Na_2_HPO_4_ buffer (pH 6.0–7.0), and 50 mM Tris-HCl buffer (pH 8.0–9.0), and incubated for 30 min at 37°C. For thermostability analysis the enzyme was incubated in 50 mM NaH_2_PO_4_-Na_2_HPO_4_ buffer (pH 7.4) at temperatures ranging from 18°C to 80°C for 30 min. The residual activity of the enzyme was then evaluated according to method adapted from Augustyniak et al. (2012), with the ortho-nitrophenyl-α-D-galactopyranoside (ONPG, Sigma, St. Louis, MO, United States) as substrate. The reaction mixture was composed of 150 μg/ml depolymerase and 3 mM ONPG in PBS in a total volume of 150 μl. After 90 min of incubation at 37°C, the absorbance at 405 nm of ortho-nitrophenol released from the chromogenic substrate was measured (Asys UVM340, Biochrom Ltd., Cambourne, United Kingdom). Relative enzyme activity was calculated and expressed as a percent reduction of absorbance compared with the control. As a control we used the depolymerase diluted in PBS, that was incubated at 4°C. The negative controls were runs with buffers and ONPG, but without the enzyme. Each experiment was performed in triplicate and repeated at least twice.

To assess the enzyme susceptibility to anionic detergent (SDS, sodium-dodecyl sulfate) and proteolytic degradation by trypsin, the samples containing 100 μg/ml of protein were incubated in the buffer (50 mM NaH_2_PO_4_-Na_2_HPO_4_, pH 7.0), and 1% (w/v) SDS (Bio-Rad, Hercules, CA, United States) or 1% (w/v) trypsin (Gibco BRL, Paisley, United Kingdom) for 10 min at RT or 100°C and at 37°C for 1 h, respectively. Further the protein samples were mixed with Laemmli buffer (Bio-Rad, Hercules, CA, United States), and analyzed with SDS-PAGE as heated and unheated samples.

### Extraction and Quantification of Capsular Polysaccharide

The total CPS was extracted from equal numbers of bacteria which were grown on TSA plates without and in the presence of enzyme for 48 h at 37°C, following the procedure of Domenico et al. (1989). Briefly, bacteria from the halo zones and lawns were scrapped from plates, washed with PBS and centrifuged (20,000 × *g*, 30 min, 4°C). The pellet was suspended in 2 ml of 0.1% Zwittergent 3–14 detergent (Calbiochem, Darmstadt, Germany) in 50 mM citric acid (pH 4.5) and heated at 42°C for 30 min. After centrifugation (17,700 × *g*, 10 min, 4°C), the supernatant was transferred to a new tube and absolute ethanol was added to a final concentration of 80% (v/v). The mixture was placed overnight at –20°C. Followed by centrifugation, the supernatant was decanted and the pellet was dissolved in 2 ml of distilled water. The CPS concentration was quantified by the uronic acid assay (Blumenkrantz and Asboe-Hansen, 1973), using a standard curve of D-glucuronic acid (Sigma-Aldrich, St. Louis, MO, United States), and expressed as μg per 10^9^ CFU.

### Serum Resistance Assay

Pooled normal human serum (NHS) was purchased from the Regional Center of Transfusion Medicine and Blood Bank (Wroclaw, Poland) and stored at −80°C. This was conducted according to the principles expressed in the Law on the public service of blood of May 20, 2016 and in the Directive 2002/98/EC of the European Parliament and of the Council of January 27, 2003, establishing standards of quality and safety for the collection, testing, processing, storage, and distribution of human blood and blood components. To determine serum sensitivity of enzyme-treated bacteria, ∼8 × 10^5^ CFU of log phase bacteria were suspended in PBS without or with the enzyme (at the final concentrations of 0.1 μg/ml, 1 μg/ml, 10 μg/ml or 100 μg/ml), and mixed at a 1:1 v/v ratio with either pooled NHS or heat-inactivated NHS (56°C, 30 min). The mixture in final volume of 200 μl was incubated for 7 h at 37°C, and at 0, 1, 2, 3, 5, and 7 h intervals, 20 μl aliquots were removed, diluted and cultured on TSA for colony enumeration. In some experiments, 10 μl of a fresh dose of depolymerase at the concentration of 1,000 μg/ml was added after 3 h and 5 h of incubation. Each test was performed at least in three independent experiments.

### Phagocytosis Assays

Phagocytosis assays were performed according to protocols adapted from Augustyniak et al. (2012, 2015) with some modifications.

#### (i) Monocyte Culture and Macrophage Differentiation

Human monocyte/macrophage cell line THP1 (ATCC, TIB-202) was maintained in RPMI-1640 medium (Lonza, Verviers, Belgium) supplemented with 10% heat-inactivated fetal bovine serum (HIFBS; GIBCO, Life Technologies, Grand Island, NY, United States), 1× glutamax (GIBCO, Life Technologies, Grand Island, NY, United States) and 1× antibiotic-antimycotic solution (GIBCO, Life Technologies, Grand Island, NY, United States) at 37°C in a 5% CO_2_ atmosphere. To induce monocyte-to-macrophage differentiation, THP-1 cells were harvested and seeded into to 96-well-flat-bottom microplates (NUNC, Thermo Fisher Scientific, Roskilde, Denmark) at density 5 × 10^5^ cells/ml (10^5^ cells per well) in complete growth medium containing 20 ng/ml of phorbol-12-myristate-13-acetate (PMA, Sigma-Aldrich, St. Louis, MO, United States). After 24 h incubation at 37°C in a humidified 5% CO_2_ atmosphere, the medium was replaced on PMA- and antibiotic-antimicotic-free medium and cells were cultured a further 24 h before use for phagocytic experiments.

#### (ii) Fluorescence Labeling of *Klebsiella* Strains

Washed bacteria (ca. 10^9^ CFU) were suspended in 200 μl of PBS without or with depolymerase (400 μg/ml). After 2 h incubation at 37°C, bacteria were harvested by centrifugation at 5,000 × *g* for 15 min at 4°C and resuspended in 1 ml of PBS. For fluorescent labeling bacteria were killed by UV for 1 h (Osram Germicidal Puritec HNS 30W G13, Russia) and subsequently incubated with 1 mg/ml of fluorescein isothiocyanate (FITC, Thermo Fischer Scientific, Rockford, IL, United States) in 0.05 M carbonate/bicarbonate buffer (pH 9.5) at 37°C for 30 min with gentle mixing in the dark. To remove the excess of dye, FITC-conjugated bacteria were washed twice with ice-cold carbonate/bicarbonate buffer, resuspended in PBS and stored at −80°C until further use.

#### (iii) Uptake of FITC-Labeled Bacteria

A 50 μl of UV-killed FITC-labeled, untreated or depolymerase-treated bacteria in Hank’s Balanced Salt Solution Ca^2+^Mg^2+^ buffer (HBSS-Ca^2+^Mg^2+^; Lonza, Verviers, Belgium) supplemented with 1% HIFBS were added to 50 μl of non-adherent THP-1 cells (10^7^ cells/ml) in the same buffer to get a MOI of 100:1. After 2 h incubation at 37°C in a humidified 5% CO_2_ atmosphere, the cells were placed on ice to halt phagocytosis and then treated with 0.2 mg/ml of trypan blue solution to quench extracellular fluorescence. As a negative phagocytic control, each bacterial strain and phagocytic cells combination was also kept on ice to block endocytic uptake of bacteria. For analysis by flow cytometry on a FACS Calibur (Becton Dickinson), the samples were diluted 1:1 in ice-cold HBSS-Ca^2+^Mg^2+^ followed by two washes in the same buffer. Each assay was performed at least three times in duplicate. The THP-1 effector cells were selected according to their forward and side scatter properties using THP-1 cells alone as a control. Data from 10,000 events (cells) per condition were collected and analyzed using Flowing Software (version 2.5.1).

#### (iv) Phagocytosis by THP-1 Differentiated Macrophages

On the day of experiments, the plates containing differentiated macrophages were washed twice with HBSS-Ca^2+^Mg^2+^ and 50 μl of untreated and depolymerase-treated bacteria in RPMI-1640 with 8% FBS was added to each well in duplicates to get a MOI of 0.1 in a final volume of 0.2 ml. The culture plates were then centrifuged at 200 × *g* for 3 min at RT to synchronize phagocytosis and incubated under 5% CO_2_ at 37°C for 2 h. After phagocytosis, infected monolayers were rinsed twice with HBSS-Ca^2+^Mg^2+^ and then incubated for additional 1 h with 200 μl RPMI-1640 containing 10% FBS, gentamicin (300 μg/ml; ICN Biomedicals, Eschwege, Germany) and polymyxin B (15 μg/ml; Sigma-Aldrich, St. Louis, MO, United States) to eliminate non-ingested bacteria. To determine the intracellular bacterial load, cells were lysed with 200 μl of 0.05% trypsin/0.08% saponin solution (v/v) for 10 min with shaking, followed by five-time washing with 200 μl of PBS each time. The lysates were then transferred to 1.5 ml Eppendorf tubes and vortexed vigorously for 30 s. The bacteria were plated in 10-fold serial dilutions onto TSA plates in triplicates and incubated overnight at 37°C. The intracellular bacterial load is presented as CFU per well. All assays were repeated at least three times. Controls were carried out by incubating bacteria in RPMI without macrophages.

### *Galleria mellonella* Killing Assay

Culture and infection of wax moth larvae (*Galleria mellonella*) with *K. pneumoniae* strains as well as the estimation of the survival rate of infected larvae was performed as outlined previously by Majkowska-Skrobek et al. (2016). Briefly, larvae were infected by injection directly into the hemocoel with 10^7^ CFUs of: (i) depolymerase-untreated bacteria, (ii) bacteria preincubated for 2 h at 37°C with depolymerase at final concentration of 200 μg/ml, and (iii) bacteria administered together with the enzyme (200 μg/ml). Three control groups were included: uninfected larvae, sham injected larvae with PBS and larvae injected with enzyme to monitor the background larval mortality, the killing due to injection trauma, and the killing due to the toxicity of the enzyme, respectively. Larvae survival was recorded for 72 h post-injection with 24 h intervals. Each test was performed in three independent experiments with 10 larvae per trial.

### Statistical Analysis

Comparisons between any two experimental groups were made by the two-sample Student’s *t*-test or, when the requirements were not met, by the Mann–Whitney *U* test. Survival curves were plotted using the Kaplan–Meier method, and the analysis in survival was performed by using the log-rank Mantel-Cox. *P* < 0.05 was considered statistically significant. All analyses were performed using GraphPad Prism software (GraphPad Software Inc.).

### Accession Number

Genome sequence of the phage KP32 (vB_KpnP_KP32) has been deposited in GenBank/EMBL under accession no. NC_013647.

## Results

### *Klebsiella* Phage KP32 Encodes Two Proteins With Capsular Polysaccharide Degrading Activity

**FIGURE 1:**
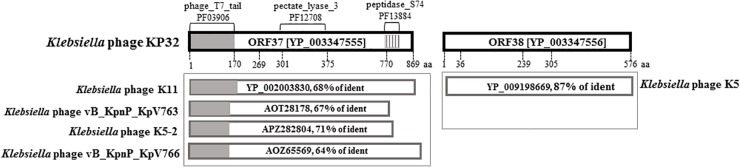
Bioinformatic sequence analysis of KP32gp37 and KP32gp38. BlastP analyses of the gp37 and gp38 gene products are presented in the boxes beneath the corresponding genes.

Protein-based screening of databases on the presence of amino acid sequence and structural homology with polysaccharide-degrading enzymes resulted in the identification of two ORFs (*gp37* and *gp38*) in *Klebsiella* phage KP32 genome, encoding a tail fiber protein and a hypothetical protein, respectively. Analysis of the *gp37* gene product (hereafter as KP32gp37) with SMART^2^ and InterProScan^3^ revealed an N-terminal phage_T7_tail domain (Pfam03906; AA: 7-150; e = 1.02e-39), pectate_lyase_3 domain (Pfam12708; AA: 301-507; e = 1.13e-05) and a C-terminal peptidase_S74 domain (Pfam13884; AA: 770-825; e = 1.49e-11) (Figure 1). The N-terminal domain exhibits high sequence similarity to the N-terminal 150 residues of other tail spikes belonging to podoviruses from “T7-like genus,” “KP32 viruses” and myoviruses that infect *Klebsiella*. Moreover, the C-terminal region has significant sequence identity to chaperone, domain of endosialidases present in the form of tail spikes or tail fibers of *E. coli*-specific phages. The protein encoded by the second ORF (KP32gp38) shows 99% similarity and 87% identity to a tail spike protein of *Klebsiella* phage K5 (YP_009198669).

In turn, a screening of the Protein Data Bank (PDB) database using HHPred and Phyre2 revealed a moderate homology of KP32gp37 to an exo-poly-α-D-galacturonosidase (PDB code 3jur), and KP32gp38 to pectin lyase-like superfamily and glycoside hydrolase family 28, enzyme groups (PDB code 1bhe) which may be both involved in polysaccharides and oligosaccharides processing.

### Biophysical and Biochemical Properties of KP32gp37 and KP32gp38 Proteins

The expressed KP32gp37 and KP32gp38 proteins were purified to electrophoretic homogeneity using two-step purification procedures (by Ni-NTA affinity and gel filtration chromatography). Purified KP32gp37 and KP32gp38 migrate as single bands on SDS-PAGE and have an estimated size of approximately 83 kDa and 62 kDa, respectively (Supplementary Figure S1A). The shorter observed length for KP32gp37 compared to predicted (Table 1) is likely due to the self-removal of the C-terminal, without affecting its activity. The purified proteins were further analyzed by SEC coupled with MALS to determine the their oligomerization state in solution. An estimated molecular weight of 259.5 ± 0.52 kDa and 179.4 ± 0.54 kDa was observed under native conditions for KP32gp37 and KP32gp38, respectively (Supplementary Figures S1B,C). Given the theoretical mass of the monomers (Table 1), confirmed under the denaturing conditions of SDS-PAGE, these findings clearly indicate a trimeric organization of both proteins. Besides the same oligomerization state, both proteins have also the same secondary structure content, as evidenced by the strong overlapping of both CD traces (Supplementary Figure S1D). The pronounced minimum at 218 nm and a positive maximum between 195 and 200 nm point to correctly folded proteins with a high β-sheet content. To gain insight into the secondary structure changes under the heat, the thermal unfolding profiles of these proteins were studied using CD spectroscopy. The thermal unfolding curve of the KP32gp37 exhibits a sigmoidal transition with a relatively high melting temperature (*T*_m_ = 74°C), while the thermal stability of KP32gp38 (*T*_m_ = 56°C) is relatively low compared to KP32gp37 (Supplementary Figure S1E).

**Table 1:** Structural and biological properties of KP32gp37 and KP32gp38.

Properties	KP32gp37	KP32gp38	Method used
Capsular-type specificity	K3	K21	Spot test
Number of amino acids	869	578	ExPASy - ProtParam tool
Theoretical pI	5.28	7.57	ExPASy - ProtParam tool
Predicted molecular mass (kDa)	95.35	61.87	ExPASy - ProtParam tool
Oligomeric state (kDa)	259.5 ± 0.52	179.4 ± 0.54	Size-exclusion chromatography coupled with light scattering
Secondary structure	Mainly β-sheet	Mainly β-sheet	CD spectroscopy
Melting temperature (*T*_m_)	74°C	56°C	CD spectroscopy
Thermostability	Up to 45°C	Up to 50°C	ONPG assay
pH stability	4–9	6–9	ONPG assay
Susceptible to SDS	Resistant	Sensitive	Electrophoresis
Susceptible to proteolysis	Resistant	Sensitive	Electrophoresis

**FIGURE 2:**
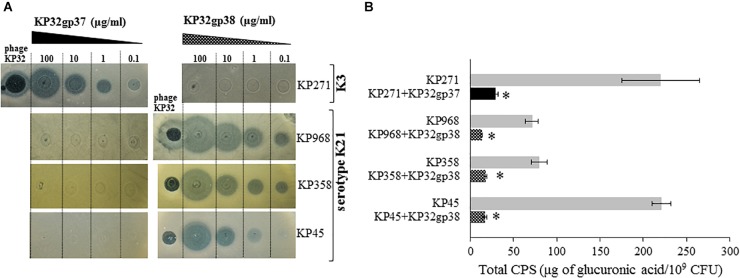
The activity of recombinant KP32gp37 and KP32gp38 proteins. **(A)** The spot test with the serial dilutions from 0.1 to 100 μg/ml of depolymerases and phage KP32 on *K. pneumoniae* (KP) strains capsular types of K3 or K21. The translucent halo zones and a clear plaque were observed after overnight incubation at 37°C when 2 μl of recombinant proteins or phage (10^6^ PFUs) were spotted on bacterial lawn, respectively. **(B)** Total CPS production by *Klebsiella* strains grown on TSA plates in depolymerase absence or presence for 48 h at 37°C, determined in the uronic acid assay. Data represents the mean of two independent experiments (mean ± SEM). Asterisks indicate statistically significant differences (^∗^*P* < 0.05) when compared with enzyme-untreated bacteria.

The activity of KP32gp37 and KP32gp38 was visualized in serial dilutions by a spot assay (Figure 2A). The translucent halo zones on the *Klebsiella* lawns following spotting of both recombinant proteins confirm their distinct enzymatic activity against different capsular types. KP32gp37 creates halo zones, even when 2 μl of enzyme (0.1 μg/ml) was dropped on a lawn of *K. pneumoniae* 271, representing capsular serotype K3. Similar halo zones were seen after KP32gp38 application on isolates corresponding to the capsular serotype K21 (strains 358 and 968). In the case of strain 45, the halo zone was visible at a 10-fold higher concentration of KP32gp38. Spot test revealed also that phage KP32 caused lytic infections and plaque formation on *Klebsiella* strains with both capsular types. Subsequently, to estimate the depolymerase-mediated reduction of the capsular material, the concentration of glucuronic acid was determined by the uronic acid assay in CPSs extracted from bacteria cultured for 2 days both in the presence and lack of the enzyme (Figure 2B). The amounts of CPS produced by the specific depolymerase-treated strains 271, 45, 358, and 968 ranged between 13.2 and 29.3 μg of glucuronic acid/10^9^ bacteria and remained 4.5- to 13-fold lower compared to the amount of CPS obtained from the equal number of untreated bacterial cells (from to 71.3 μg/10^9^ cells to 221 μg/10^9^ cells, *P* < 0.05).

Beyond the confirmation of their biological activity, KP32gp37 and KP32gp38 have been studied in detail for their stability at various pH values and temperatures, tolerance to anionic detergent, and resistance to proteolytic degradation. Both enzymes revealed an optimal stability under slightly alkaline conditions from pH 7.0 to 9.0, maintaining 74.8 ± 0.8% to 100.0 ± 0.6% their activity, respectively (Supplementary Figure S2A). After an incubation for 1 h under acidic conditions (pH 6.0 and below), less than 30% of their original activity was preserved. In addition, both enzymes remain active over the temperature range from 18°C to 45°C, maintaining more than 95% of their activity (Supplementary Figure S2B). At 50°C KP32gp38 displayed more than 90% of its activity, while the KP32gp37 activity dropped to 16.7 ± 1.1%.

Based on electrophoretic mobility of proteins, it has been shown that these depolymerases differ in sensitivity to both anionic detergent and protease. KP32gp37 is resistant against denaturation by SDS at room temperature and against digestion by trypsin (Supplementary Figure S2C). By contrast, KP32gp38 was found to be sensitive to both SDS-induced denaturation and proteolytic cleavage. Table 1 summarizes biochemical properties and structural features in solution of both depolymerases.

### Depolymerase Influence on *K. pneumoniae* Sensitivity to Complement-Mediated Killing

**FIGURE 3:**
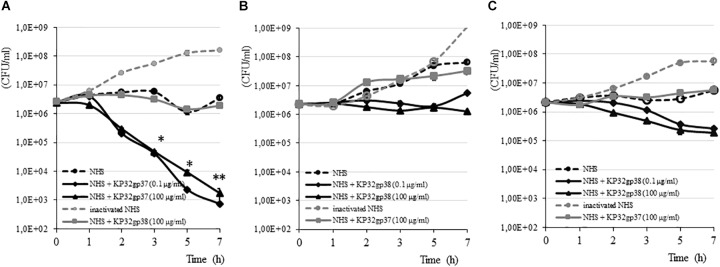
Time-dependent bactericidal effect of serum against *K. pneumoniae* strains 271 **(A)**, 45 **(B)** and 358 **(C)** treated and untreated with a capsule serotype-specific and non-specific depolymerase. Bacteria were grown in 50% NHS or heat inactivated NHS, in the presence or absence of depolymerases for 7 h at 37°C. Serially diluted aliquots were plated on TSA plates. The results were expressed as mean ± SEM. As the survival of *Klebsiella* strains treated and untreated of a capsule serotype-specific enzyme in the presence of heat inactivated NHS was comparable, so for simplicity, only one of these data has been shown. Asterisks indicate statistically significant differences (^∗^*P* < 0.05, ^∗∗^*P* < 0.001) compared to the initial inoculum. For each option, at least three independent experiments were performed.

The susceptibility of *K. pneumoniae* to the bactericidal activity of serum depends on the structure and organization of both CPS and the LPS O-side chain (Álvarez et al., 2000; Cortés et al., 2002). Of the four clinical *Klebsiella* strains analyzed by us, three were characterized as serum resistant showing a survival of 100% or even a propagation event after 7 h of incubation in 50% NHS (Figure 3). *Klebsiella* strain 968 was sensitive to NHS killing with a reduction of CFU in the range of six orders after 30 min of incubation (data not shown). To define the influence of depolymerase-induced degradation of CPS on the survival of serum-resistant strains, the complement bactericidal assays were performed in the presence of enzymes. The treatment of strains with a capsule serotype-specific enzyme produced a time-dependent decline in survival, while bacteria treated with non-specific enzyme (100 μg/ml) or incubated only in the presence of NHS were protected from the lytic effect of complement. For K3-type strain (271), 0.1 μg/ml of KP32gp37 caused a decrease of bacterial number, respectively, by 1.6 order of magnitude upon 3 h exposure (from 2.54 × 10^6^ ± 4.80 × 10^4^ CFU/ml to 4.04 × 10^4^ ± 6.90 × 10^3^ CFU/ml, *P* < 0.05) and almost four orders upon 7 h (from 2.54 × 10^6^ ± 4.80 × 10^4^ CFU/ml to 7.33 × 10^2^ ± 8.82 × 10^1^ CFU/ml, *P* < 0.001) (Figure 3A). Increasing KP32gp37 concentration to 100 μg/ml did not significantly affect the sensitivity of bacteria to the bactericidal action of serum. In contrast, K21-type strains showed only 1.7- to 4.6-fold CFU reduction compared to the initial inoculum upon 3 h exposure to KP32gp38 at 100 μg/ml in the presence of NHS (from 2.22 × 10^6^ ± 8.66 × 10^4^ CFU/ml to 1.34 × 10^6^ ± 1.49 × 10^5^ CFU/ml for strain 45 and from 2.16 × 10^6^ ± 1.0 × 10^5^ CFU/ml to 4.86 × 10^5^ ± 5.30 × 10^4^ CFU/ml for strain 358), (Figures 3B,C). The prolongation of the incubation time up to 7 h resulted in a further gentle CFU reduction of strain 358 (to 1.87 × 10^5^ ± 3.13 × 10^4^ CFU/ml), while the strain 45 has not increased its susceptibility to complement action, even if the fresh doses of KP32gp38 were added after 3 and 5 h. The bacterial number of strain 45 remained at the level of 1.26 × 10^6^ ± 2.34 × 10^5^ CFU/ml, whereas after the administration of two additional doses of enzyme was 7.52 × 10^5^ ± 9.85 × 10^4^ CFU/ml. When bacteria were incubated with or without enzyme in the presence of heat-inactivated NHS, no killing was observed for any of tested strains, suggesting that the killing was effectively due to the lytic action of complement. These findings indicate that the increase in complement activity against depolymerase-treated *Klebsiella* cells is corresponding to the serotype-related variations of CPS structure.

### Exposure to Depolymerase Sensitizes *Klebsiella* Cells to Phagocytosis

**FIGURE 4:**
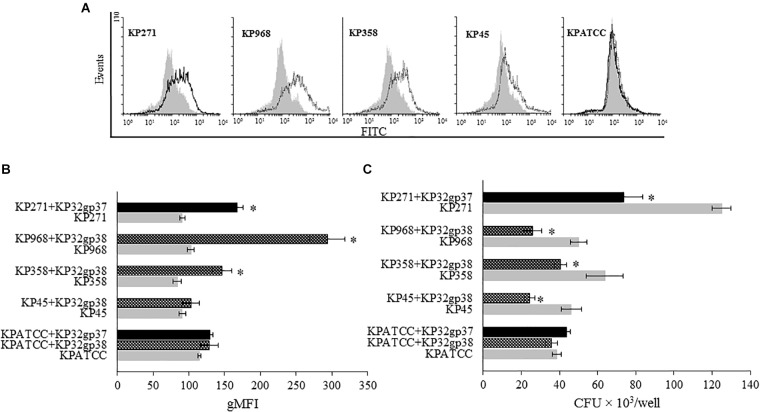
Depolymerase-mediated phagocytosis of *K. pneumoniae* strains by the human monocyte cell line THP1 determined by flow cytometry **(A,B)** and plate counting **(C)**. Representative flow cytometric plots: **(A)** histograms depict increased uptake of depolymerase-treated bacteria vs. untreated bacteria. UV-killed FITC-labeled *K. pneumoniae* (KP) strains 271, 358, 968, 45 and ATCC (non-K3/K21 serotype used as a control), preincubated or not with corresponding depolymerase, were mixed with monocytes at 100:1 ratio. Black peaks show the log of fluorescence intensity of monocytes exposed to depolymerase-treated *K. pneumoniae* strains; gray peaks depict log fluorescence intensity of monocytes exposed to untreated bacterial cells. **(B)** Quantification of ingested depolymerase-treated or untreated bacteria labeled with FITC by monocytes as measured by flow cytometry and analyzed using Flowing Software. Data are expressed as the geometric mean fluorescence intensity (gMFI) from the gated sample with phagocytozing cells ± SEM. Results are representative of three independent experiments. **(C)** Quantification of intracellular bacteria in THP1-differentiation macrophages infected with *Klebsiella* cells in the present or without corresponding depolymerase. The number of surviving bacteria was determined by macrophage lysis and colony count. Data represent the mean ± SEM of CFU × 10^3^/well from three independent experiments performed in duplicate (*n* = 6). Asterisks indicate statistically significant differences (^∗^*P* < 0.05) when compared to corresponding controls (no depolymerase, gray bars).

To study whether the depolymerase-induced degradation of bacterial CPS affects phagocytosis by THP1 cells, we used two different methods. The first one was flow cytometric analysis in which the quantitative uptake of UV-killed FITC-labeled *K. pneumoniae* strains by monocytes, was determined. Untreated or enzyme-treated bacterial cells were compared. The second method was the classical bacteriological method, in which THP1 differentiated macrophages infected with bacteria were lysed for intracellular bacterial quantification. The geometric Mean Fluorescence Intensity (gMFI) of monocytes after a 120-min incubation with enzyme-treated bacteria, at an infection ratio of 100 bacteria per cell, was significantly higher than of cells incubated with untreated bacteria (*P* < 0.05), (Figures 4A,B). KP32gp37 enhanced the uptake of strain 271 nearly twofold, from 91.0 ± 3.5 gMFI for untreated to 167.3 ± 8.3 gMFI for enzyme-treated bacteria. Similar results were obtained for the uptake of two strains possessing degraded capsules by the second depolymerase. KP32gp38 treatment increased the uptake of strain 358 from 83.9 ± 5.7 gMFI to 146.7 ± 13.6g gMFI, whereas these values for strain 968 were 102.3 ± 6.8 gMFI and 293.6 ± 24.4 gMFI, respectively. Likewise, when *K. pneumoniae* cells were phagocytized by THP1 differentiated macrophages in the presence of serotype-specific depolymerase, their intracellular bacterial load was considerably reduced (up to 52%) compared to enzyme-untreated bacteria. That proves the effective killing of pathogens with degraded capsular layer, especially when considering that a higher number of cells is taken up when the capsule is depolymerized (Figure 4C). These results suggest that depolymerases, likely by uncovering ligands for phagocytic cell attachment, can defeat the resistance of encapsulated bacteria to phagocytosis and sensitize them to the uptake and killing in this process.

### Depolymerase Application Prolongs the Survival of *Klebsiella* Infected Wax Moth Larvae

**FIGURE 5:**
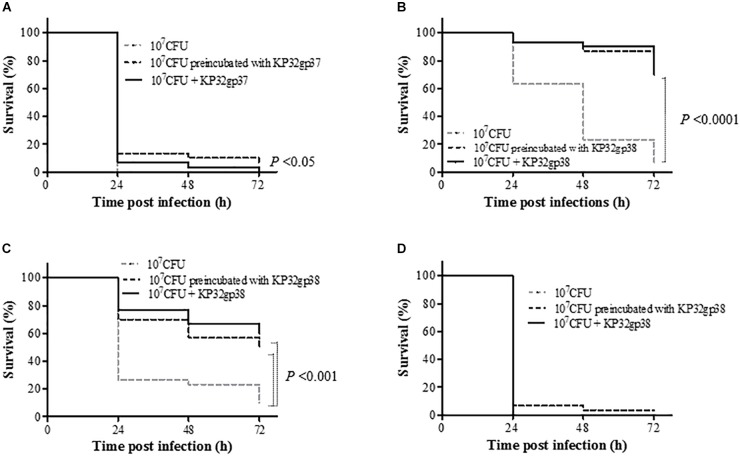
Kaplan–Meier survival curves following injection of *G. mellonella* larvae with *K. pneumoniae* strains **(A)** 271, **(B)** 968, **(C)** 358 and **(D)** 45. Larvae were injected with either bacteria (10^7^ CFUs/per larvae), bacteria preincubated with serotype-specific depolymerase or enzyme administrated simultaneously with bacteria. Larvae survival was monitored for 3 days post injection. The experiments were controlled by uninfected larvae, PBS-injected larvae and larvae receiving the enzyme only. Survival for each control group was 100%, so for simplicity, these groups were not included in the figure. Data represent three independent experiments with a total of 30 larvae per bacterial strain. Statistically significant differences in survival were calculated by the log-rank test.

As the immune system of *G. mellonella* larvae in many aspects is remarkably similar to the innate immune response of the higher organisms to *Klebsiella* infections (Insua et al., 2013), we used the insect model to evaluate the virulence of depolymerase-preincubated bacterial cells as well as the efficacy of treatment with these enzymes *in vivo*. For three out of four tested *Klebsiella* strains, an injection of bacteria pretreated with a capsule serotype-specific depolymerase as well as a single dose of the enzyme administered simultaneously with bacteria significantly affected the larval survival rate compared to the bacteria injected groups (*P* < 0.05) (Figure 5). The anti-K3 depolymerase (KP32gp37) was able to prolong the larvae survival of at least 13% after 24 h post-infection and the treatment exposition profiles were similar to each other. The application of KP32gp38 was very effective on two K21-strains (358 and 968) regardless of the type of infection treatment (preincubated bacteria or bacteria plus enzyme). In this case, the survival rates of larvae were at least 30% higher comparing to the control after 24 h post-infection. Moreover, at least 50 and 70% of larvae inoculated 358 and 968 strains, respectively, were alive at the end of the experiment (72 h) (*P* < 0.001). Unexpectedly, the K21-strain 45 turned out to be *in vivo* almost fully insensitive to depolymerase activity. No mortality of larvae was observed in the control groups, upon injection of PBS buffer or enzyme alone. In general, we may state that depolymerase treatment can rescue larvae against bacterial infection indicating that these enzymes are able to attenuate the *Klebsiella* virulence in a time- and strain dependent manner.

## Discussion

Capsule, a major factor for *Klebsiella* virulence and phage susceptibility, is a crucial element in the phage-bacterium-host interplay. In the present study, we have evaluated two newly characterized depolymerases derived from *Klebsiella* phage KP32 (KP32gp37 and KP32gp38), exhibiting the capacity to recognize and digest the capsules from strains representing K3 and K21 serotypes, respectively. We addressed two major questions: (i) can these phage-encoded enzymes counteract the resistance of encapsulated *K. pneumoniae* to key mechanisms of the innate host defense such as complement-mediated lysis and phagocytosis by macrophages, and (ii) if so, what is the role of the capsular serotype and depolymerase specificity? We have demonstrated that depolymerase-induced capsule degradation, effectively disarms the pathogen, resulting in complement-mediated killing, increased phagocytosis and killing by macrophages. In addition, the depolymerases are highly specific for a particular capsule serotype. Considering the potential of these phage-encoded proteins as antivirulence compounds, we also determined biochemical and biophysical properties of both enzymes.

In our study, the specificities of KP32gp37 and KP32gp38 against encapsulated bacteria were validated based on *in vitro* spot assays and quantitative determination of glucuronic acid in capsular material extracted from bacteria growing in the presence of enzyme. In contrast to phage KP32 that causes lytic infection and halo zone formation on bacterial lawns of both K3 and all K21 strains, each of the proteins possesses CPS depolymerization activity toward only one capsular serotype. The presence of two CPS depolymerases in the KP32 genome thus expands the host spectrum of phage KP32.

The capacity of *K. pneumoniae* to circumvent the immune response mechanisms ensures the supremacy of the pathogen over the host and contributes to a successful infection process. Previous studies pointed to at least two mechanisms that contribute to capsule-mediated resistance to complement action. The first mechanism is the masking effect of CPS on bacterial molecules that act as targets for complement activation and deposition. These molecules include: (i) the epitopes on the bacterial surface that are necessary for classical pathway (CP) activation in an antibody-dependent manner, (ii) the OMPs that can trigger the CP in antibody-independent manner upon interaction with C1q component, (iii) LPS that allows the deposition of C3b in three activation pathways (Merino et al., 1992; Albertí et al., 1993, Albertí et al., 1996; Doorduijn et al., 2016). The second mechanism is correlated to the serotype-related differences in capsular composition and the ability of some strains to modify the polysaccharide backbone. It was shown that MBL fail to recognize CPS deprived of mannobiose or rhamnobiose repeating units, thereby inhibiting the lectin pathway (LP), with the subsequent reduced deposition of C3b and cell lysis (Sahly et al., 2009).

Our results showed that in response to the bactericidal action of serum, the number of KP32gp37-treated K3 bacteria was significantly decreased compared with non-treated or bacteria incubated with or without enzyme in the presence of heat-inactivated NHS. The survival of KP32gp38-treated K21 *Klebsiella* strains merely decreased a 2- to 11-fold. However, the remaining amounts of CPS present in depolymerase-treated bacteria were an average sixfold lower than those of their encapsulated parental strains and maintained at a similar level, regardless of the enzyme serotype specificity. Analyzing above results we may suspect that only following CPS degradation of K3-type, the complement components gained access to activators on the cell surface (porins and rough LPS), leading to increased deposition of C3b on-serum resistant cells and the C5-9 MAC formation, which resulted in killing of pathogen. In general it might be concluded that *Klebsiella* capsular serotype is more important for protection against complement-mediated killing than the amount of CPS produced by cells following depolymerase treatment. The repeating units of both K3 and K21 CPSs are the pyruvic acetal-bearing pentasaccharide containing mannose, galactose, and glucuronic acid in a ratio of 3:1:1 and 2:2:1, respectively (Dutton and Choy, 1972; Dutton et al., 1986). Thus, even slight differences in capsule sugar composition determine bacterial susceptibility to phage-derived depolymerase. This unique specificity of the enzymes allows them to be used in targeted therapy against *Klebsiella* strains with defined serotype. On the other hand, it is possible to extend the substrate spectrum for such enzymes (Simpson et al., 2015). It was shown that both changing of residues in the active site by mutation and swapping with foreign domains, already active on a selected polysaccharide, enables to generate enzymes with changed substrate specificity (Barbirz et al., 2008; Leiman and Molineux, 2008). It is worth noting that, despite the presence of mannobiose participating in LP activation, serum-resistance of all strains tested in this work (except for strain 968) suggests that this pathway is not a dominant or sufficient to kill them by complement.

To further characterize the innate response against depolymerase-treated bacteria, we assessed their capacity to trigger non-opsonic (lectino-)phagocytosis. Unlike the observed differences between serotypes in terms of their ability to resist complement following depolymerase treatment, such dependency was not seen for the susceptibility to phagocytosis. We proved that UV-killed depolymerase-treated bacteria of both serotypes were more effectively taken up by THP1 monocytes than the unmodified parent bacteria, excluding strain 45. Also the number of the bacterial cells killed by THP1 monocyte-derived macrophages was significantly higher after modification of their phenotype by capsule-specific enzymes. Depolymerase-mediated, enhanced uptake may be explained by the nature of the sugar residues present in CPS. Some *K. pneumoniae* serotypes display surface glycans with terminal mannose residues that are recognized by the mannose receptor of macrophages, thereby acting as a marker of non-self (Wileman et al., 1986; Athamna et al., 1991; Sahly et al., 2008). Upon recognition, the receptor internalizes the bound pathogen and transports it to lysosome for degradation via the phagocytic pathway. The strains of both serotypes studied in this work (K3 and K21) possess internal mannosyl residues in their CPS structures (Dutton and Choy, 1972; Dutton et al., 1986). Thus, the increased susceptibility to phagocytosis after depolymerase treatment may be explained by a concomitant increased exposure of previously inaccessible mannose residues to the macrophage receptors. On the other hand, the increased uptake of depolymerase-treated cells by monocytes, which do not express the mannose receptor (Shepherd et al., 1982), indicates that other receptors or mechanisms may be involved in this process. Therefore, even for strains bearing glyco-epitopes in their capsule promoting lectinophagocytosis, enzymatic removal of CPS barrier improves this process, probably by an appropriate presentation and accessibility of ligands to phagocytic cell receptors.

An important question is whether *in vivo* capsule removal by phage-derived depolymerase would improve the susceptibility of bacteria to the host’s immune response. Lin et al. (2014) demonstrated that intraperitoneal administration of recombinant depolymerase encoded by a K1-specific phage, NTUH-K2044-K1-1, significantly increased the survival rate of mice pre-treated with cyclophosphamide and then infected with *K. pneumoniae* NTUH-K2044. Similar therapeutic efficacy of a K64 specific depolymerase has been shown in cyclophosphamide-treated mice infected with multidrug-resistant *K. pneumoniae*, although this enzyme was ineffective if administrated 8 h post-infection (Pan et al., 2015). Given that cyclophosphamide reduce leucocytes counts without affecting the level of serum complement (Li et al., 1987), these observations suggest that complement-dependent mechanism is responsible for clearance of enzyme-treated bacteria and protection for infection. Also in our study, it has been demonstrated a significant prolongation of the larvae lifespan following the infection of bacteria preincubated with a capsule serotype-specific depolymerase or an application of enzyme to treat infected caterpillars. It proves that these phage-derived proteins are able to reduce *Klebsiella* virulence in *in vivo* model. Nevertheless, for some strains (*Klebsiella* 45 of K21 serotype) the degradation of capsule by phage-derived depolymerase did not significantly affect the phagocytic uptake implicating further unsuccessful infection treatment in moth larvae model.

Exposed on the surface of the virion, tail spikes or fibers bind specific sequences of carbohydrates to recognize the bacterial host cell (Drulis-Kawa et al., 2015), thus their structures must be stable to oppose the harsh environmental conditions, i.e., high temperatures, non-physiological pH, the presence of extracellular proteases, etc., to keep phage infectivity (Barbirz et al., 2008). Many tail spikes and fibers obtain stability through the formation of a trimeric β-structure, resembling a puncturing needle (Steinbacher et al., 1994; Kanamaru et al., 2002; Weigele et al., 2003). In addition to trimer formation, other attractive properties of both tested proteins are their moderate thermostability up to 74°C and 56°C and maximum activity under slightly alkaline conditions in the range pH values from 7.0 to 9.0, similar to most *Klebsiella* phage-derived depolymerases (Bessler et al., 1973; Kassa and Chhibber, 2012). In contrast, these enzymes can be differentiated in terms of resistance to anionic detergent and proteolytic cleavage.

## Conclusion

We demonstrated that two depolymerases encoded by *Klebsiella* phage KP32 degrading capsules of K3 and K21 serotypes, are able to sensitize bacteria to serum complement lysis, increase an uptake and killing of *K. pneumoniae* by macrophages in the lectinophagocytosis process as well as are effective in *in vivo* infection model in a serotype- and strain-dependent manner. In general, these enzymes have beneficial properties for its further application in therapy or for biotechnology purposes.

## Author Contributions

AL, YB, RB, FS, and GM-S designed and carried out the experiments needed to produce protein and characterize its structural features in solution. BM and GM-S designed and performed definitive identification the capsular type of *Klebsiella* isolates. GM-S designated and performed the studies evaluating both the enzymes stability and activity *in vitro* and *in vivo*, conducted statistical analyses, and wrote the manuscript. ZD-K, GM-S, RB, and YB conceived the research, interpreted the data, and revised the manuscript. All authors read and approved the final manuscript.

## Conflict of Interest Statement

The authors declare that the research was conducted in the absence of any commercial or financial relationships that could be construed as a potential conflict of interest.

## Footnotes

1 http://web.expasy.org/protparam

2 http://smart.embl-heidelberg.de

3 http://www.ebi.ac.uk/Tools/pfa/iprscan/
