# Supplementary material for: Phage-Borne Depolymerases Decrease Klebsiella pneumoniae Resistance to Innate Defense Mechanisms
Source: Front Microbiol. 2018 Oct 23;9:2517. doi: 10.3389/fmicb.2018.02517 (PMC6205948; doi:10.3389/fmicb.2018.02517)
Supplement: Supplementary file 1 [file Data_Sheet_1.PDF]

Supplementary Material

Phage-borne depolymerases decrease *Klebsiella pneumoniae* resistance to innate defense mechanisms

Grazyna Majkowska-Skrobek[1*], Agnieszka Latka[1,2], Rita Berisio[3], Flavia Squeglia[3], Barbara Maciejewska[1], Yves Briers[2] and Zuzanna Drulis-Kawa[1*]

*** Correspondence:** Grazyna Majkowska-Skrobek: grazyna.majkowska-skrobek@uwr.edu.pl and Zuzanna Drulis-Kawa: zuzanna.drulis-kawa@uwr.edu.pl

Supplementary Table S1. Bacterial strains and primers used in this study

Strains and primers	Description or sequence (5'→3')	Source and/or reference
**Strains**		
***K. pneumoniae* 700603**	capsular type non-K3/K21 serotype	American Type Culture Collection
***K. pneumoniae* 271**	clinical isolate, capsular type K3 serotype	Institute of Genetics and Microbiology Bacterial Collection (Wroclaw, Poland)
***K. pneumonaie* 45**	clinical isolate, capsular type K21 serotype	
***K. pneumoniae* 358**	clinical isolate, capsular type K21 serotype	
***K. pneumoniae* 968**	clinical isolate, capsular type K21 serotype	
**Primers for cloning**		
**KP32gp37-F**	ATGGACCAAGATATTAAAACAATCAT TCAGTAC	this study
**KP32gp37-R**	TTATTTGTAGGTCAGGCCGAGCTTACG	
**KP32gp38-F**	ATGTTAGACAATTTCAATCAGCCG	
**KP32gp38-R**	TGATACGAATGCCCTTACTCGG	
**Primers for typing**		
**WZC_CR1**	TTCAGCTGGATTTGGTGG	(Pan et al., 2013)
**WZA_CF1**	TGAAAGTGTTTGTCATGGG	

Figure supplementary legends

Supplementary Figure S1. Structural characterization of KP32gp37 and KP32gp38 in solution. (A) SDS-PAGE profile of recombinant proteins after Ni-NTA affinity column purification. MW,

molecular mass markers; lanes 1 and 2, Commassie blue stained purified proteins. **(B)** and **(C)** SEC–MALS analyses determining the oligomerization state and molar mass of KP32gp37 and KP32gp38, respectively. The curves depict the Rayleigh ratio (left scale) of proteins versus the retention time. Lines under the peaks correspond to the averaged molecular mass (right-hand y axis) distributions across the peak as determined by MALS. **(D)** CD spectra and **(E)** melting curves demonstrating the stability of depolymerases. CD spectra were recorded (0.25 mg/ml) in 20 mM sodium phosphate buffer (pH 6.0 for KP32gp37 and pH 7.4 for KP32gp38) at 20°C. Thermal denaturation curves were measured at 214 nm. The midpoint of each curve was used to calculate the melting transition temperature (Tm) for proteins. Data for KP32gp37 and KP32gp38 are marked with the same colour code.

Supplementary Figure S2. Stability of KP32gp37 and KP32gp38. **(A)** Effect of pH and **(B)** various temperatures at pH 7.4 on the ONPG-degrading activity of depolymerases. The optimal pH was determined at 37°C in 50 mM CH_3COONa-HCl buffer (pH 3.0 to 5.0), 50 mM NaH_2PO_4-Na_2HPO_4 buffer (pH 6.0 to 7.0), and 50 mM Tris-HCl buffer (pH 8.0 to 9.0). Relative enzyme activity was calculated and is expressed as a percent reduction of absorbance compared with control without enzyme. Each experiment was performed in triplicate and repeated at least twice. The data represent means ± SD. **(C)** KP32gp37 (upper panel) and KP32gp38 (bottom panel) susceptibility to denaturation in the presence of 1% SDS and proteolysis. Lane MW: molecular weight markers, lanes: (1) protein boiled, (2) protein non-boiled, (3) protein + trypsin, boiled, (4) protein + trypsin, non-boiled, (5) BSA boiled, (6) BSA + trypsin, boiled, (7) trypsin boiled.
