# Supplementary material for: Phage-Borne Depolymerases Decrease Klebsiella pneumoniae Resistance to Innate Defense Mechanisms
Source: Front Microbiol. 2018 Oct 23;9:2517. doi: 10.3389/fmicb.2018.02517 (PMC6205948; doi:10.3389/fmicb.2018.02517)
Supplement: Supplementary file 2 [file Data_Sheet_2.PDF]

SUPPLEMENTARY FIGURE S1

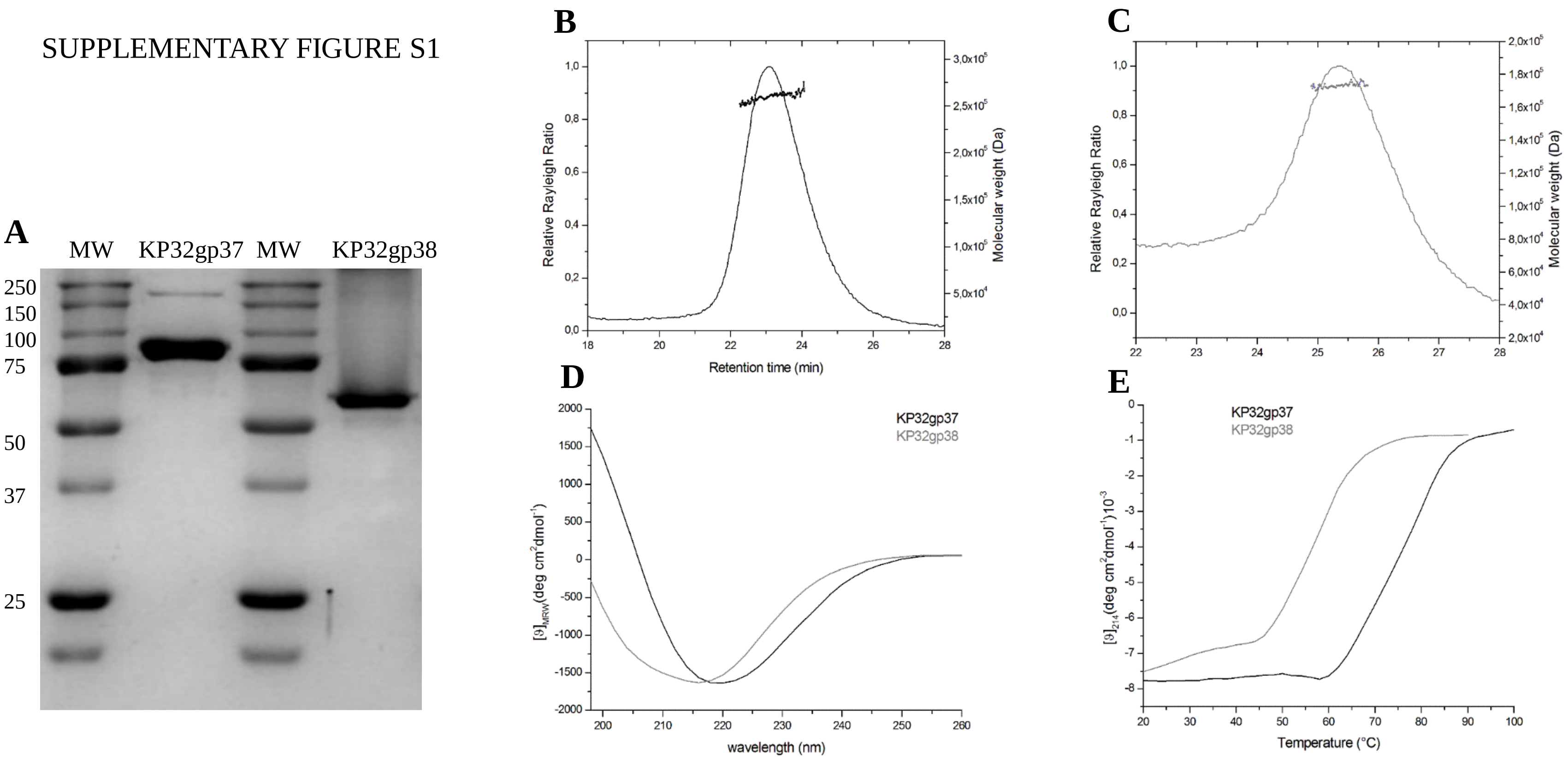

Figure S1. Structural characterization of KP32gp37 and KP32gp38 in solution. (A) SDS-PAGE profile of recombinant proteins after Ni-NTA affinity column purification. MW, molecular mass markers; lanes 1 and 2, Commassie blue stained purified proteins. **(B)** and **(C)** SEC–MALS analyses determining the oligomerization state and molar mass of KP32gp37 and KP32gp38, respectively. The curves depict the Rayleigh ratio (left scale) of proteins versus the retention time. Lines under the peaks correspond to the averaged molecular mass (right-hand y axis) distributions across the peak as determined by MALS. **(D)** CD spectra and **(E)** melting curves demonstrating the stability of depolymerases. CD spectra were recorded (0.25 mg/ml) in 20 mM sodium phosphate buffer (pH 6.0 for KP32gp37 and pH 7.4 for KP32gp38) at 20°C. Thermal denaturation curves were measured at 214 nm. The midpoint of each curve was used to calculate the melting transition temperature (Tm) for proteins. Data for KP32gp37 and KP32gp38 are marked with the same colour code.
