# Supplementary material for: Phage-Borne Depolymerases Decrease Klebsiella pneumoniae Resistance to Innate Defense Mechanisms
Source: Front Microbiol. 2018 Oct 23;9:2517. doi: 10.3389/fmicb.2018.02517 (PMC6205948; doi:10.3389/fmicb.2018.02517)
Supplement: Supplementary file 3 [file Data_Sheet_3.PDF]

SUPPLEMENTARY FIGURE S2

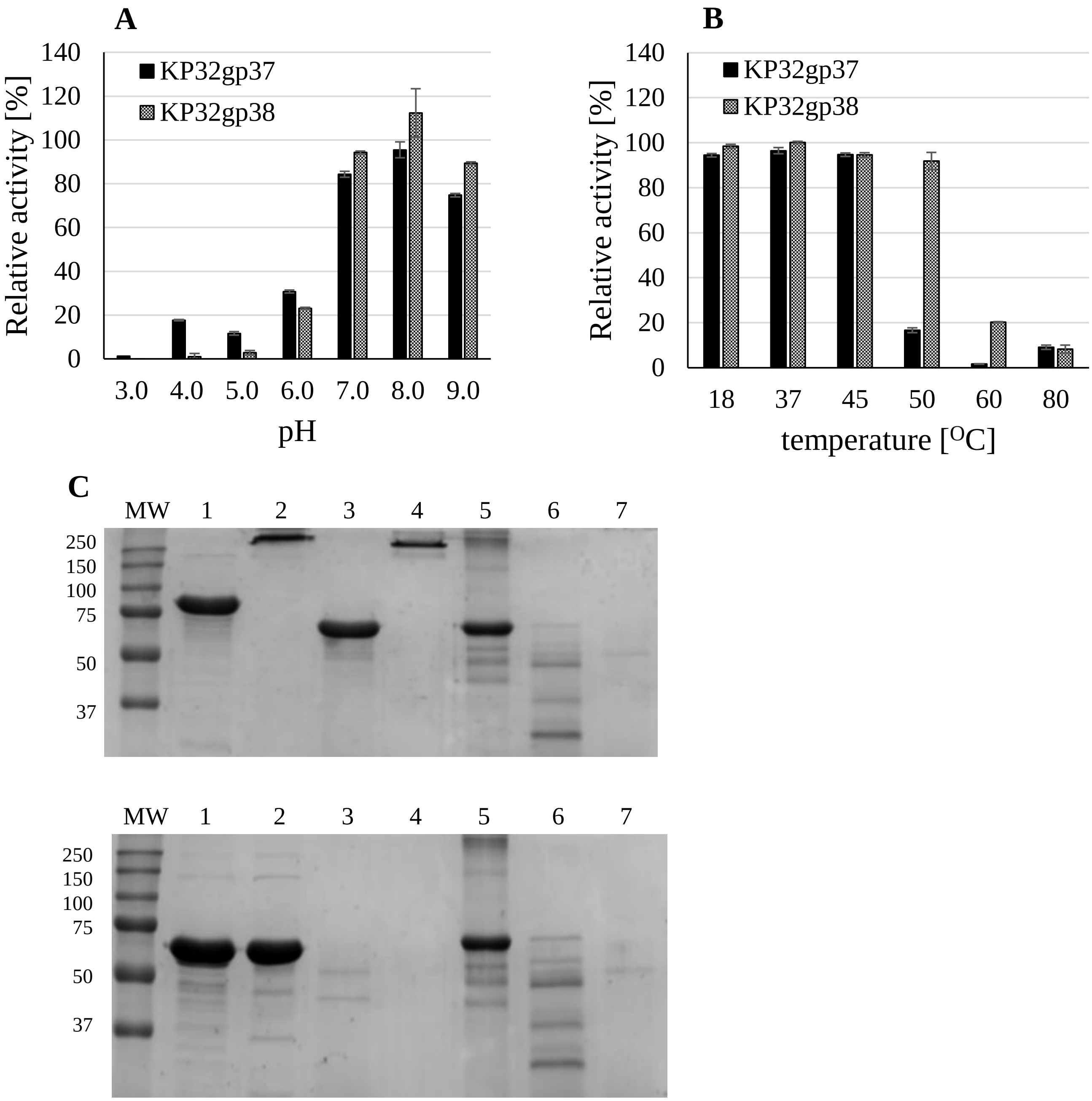

Figure S2. Stability of KP32gp37 and KP32gp38. **(A)** Effect of pH and **(B)** various temperatures at pH 7.4 on the ONPG-degrading activity of depolymerases. The optimal pH was determined at 37°C in 50 mM CH_3COONa-HCl buffer (pH 3.0 to 5.0), 50 mM NaH_2PO_4-Na_2HPO_4 buffer (pH 6.0 to 7.0), and 50 mM Tris-HCl buffer (pH 8.0 to 9.0). Relative enzyme activity was calculated and is expressed as a percent reduction of absorbance compared with control without enzyme. Each experiment was performed in triplicate and repeated at least twice. The data represent means ± SD. **(C)** KP32gp37 (upper panel) and KP32gp38 (bottom panel) susceptibility to denaturation in the presence of 1% SDS and proteolysis. Lane MW: molecular weight markers, lanes: (1) protein boiled, (2) protein non-boiled, (3) protein + trypsin, boiled, (4) protein + trypsin, non-boiled, (5) BSA boiled, (6) BSA + trypsin, boiled, (7) trypsin boiled.
